# Effects of herbal supplements on milk production quality and specific blood parameters in heat-stressed early lactating cows

**DOI:** 10.3389/fvets.2023.1180539

**Published:** 2023-06-02

**Authors:** Ahmed Ali Saleh, Mahmoud Mohamed Soliman, Mohammed Farid Yousef, Nabil Mohammed Eweedah, Hanan Basiouni El-Sawy, Mustafa Shukry, Mohammad A. M. Wadaan, In Ho Kim, Sungbo Cho, Hossam M. Eltahan

**Affiliations:** ^1^Department of Poultry Production, Faculty of Agriculture, Kafrelsheikh University, Kafr El-Sheikh, Egypt; ^2^Departments of Animal Production, Faculty of Agriculture, Kafrelsheikh University, Kafr El-Sheikh, Egypt; ^3^Department of Nutrition and Clinical Nutrition, Faculty of Veterinary Medicine, Kafrelsheikh University, Kafr El-Sheikh, Egypt; ^4^Department of Physiology, Faculty of Veterinary Medicine, Kafrelsheikh University, Kafrelsheikh, Egypt; ^5^Department of Zoology, College of Science, King Saud University, Riyadh, Saudi Arabia; ^6^Animal Resource and Science Department, Dankook University, Cheonan, Republic of Korea; ^7^Animal Production Research Institute, Agriculture Research Center, Ministry of Agriculture, Dokki, Egypt

**Keywords:** herbal mixture, Ovum, dairy cows, fatty acids, milk production

## Abstract

The present study explored the influence of supplemental herbal mixtures on cow milk production, quality, and blood parameters in dairy cows under high ambient temperatures. Thirty Holstein cows were randomly assigned into three experimental groups of 10 each. The first control group was supplied with the commercial basal diet, whereas two treatment groups were provided with the commercial basal diet supplemented with 50 and 100 g/head/day of the herbal mixture, respectively. The results showed that the mixture of herbal supplementation did not influence weekly milk production. Milk total fat, triglyceride, and total protein values were not affected (*p* < 0.05) in cows fed on basal diets supplemented with herbal mixture; however, milk cholesterol was decreased significantly by 100 mg/head/day of the herbal mixture. On the other hand, lactose has increased significantly by adding 100 mg/head/day of herbal mixture. Furthermore, the total cholesterol level in serum was decreased by adding 100 mg/head/day of the herbal mixture, while plasma prolactin, cortisol, GOT, and GPT were unaffected. Regarding fatty acids (C18, C18:1 (c9), 18:1 (c11), 18:2 (c9, c12), 18:2 (t9, t12), and CLA (c9, t11)), there was no significant variation between the groups. Meanwhile, both C19:00 and 18:3 (c6, c9, and c12) were noticeably higher (*p* < 0.05) in the group that received 100gm, followed by 50 mg, compared to the control. In conclusion, the supplement with a herbal mixture positively affected milk quality by decreasing total cholesterol and increasing lactose, milk fatty acid profile by increasing unsaturated fatty acids content, and plasma cholesterol levels.

## 1. Introduction

Globally, around 150 million farmers are engaged in milk production. In the majority of developing nations, smallholders produce milk, which contributes to household livelihoods, food security, and nutrition. Recently, developing nations have expanded their proportion of global dairy production (1). This expansion is primarily attributable to an increase in the number of producing animals rather than an increase in productivity per head. In numerous developing nations, dairy production is stifled by low-quality feed resources, illnesses, limited access to markets and services (e.g., health, credit, and training), and the low genetic potential of dairy animals. Many emerging nations, unlike developed countries, have hot and/or humid conditions that are adverse for dairy farming (2, 3).

The paramount necessity for dairy economics is optimizing both milk production and quality. Effective ecological farming methods are required due to the rapidly rising global demand for dairy products, particularly in developing nations, and growing environmental concerns (4). However, compared to the worldwide average, milk yield per cow remains quite low in developing nations (5, 6).

Various medications, herbal remedies, hormones, mineral supplements, and feed additives have already been attempted to boost milk production and animal productivity in specific animals (7–10). However, most of these herbal remedies have not undergone extensive scientific evaluation, despite their long-standing use raising particular safety and efficacy concerns. Numerous variables could explain this lower yield (11).

Plants contain a wide variety of secondary metabolites that, when concentrated and extracted, may have antibacterial effects on rumen microorganisms (12, 13). Plant secondary metabolites have been thoroughly assessed for their potential to influence ruminal fermentation, enhance ruminant nutrition use, and their antibacterial activities (14). Several studies have been devoted to assessing the possible use of plant extracts as antibiotic replacement feed for ruminants. Herbs or botanicals could boost feed intake and digestive juice production, stimulating the immune system and possessing antimicrobial properties. The endocrine system and the metabolism of intermediate nutrients can be stimulated by herbs, which can also help meet the nutritional needs of animals (15, 16). For example, Ovuma is a powerful herbal formulation containing 98% betaine, fenugreek, flaxseed, curcumin, and peppermint leaves. Betaine (trimethylglycine) is widespread in animals, plants, and microbes (17). Recent research indicates that betaine mitigates oxidative damage in heat-stressed bovine mammary epithelial cells (BMECs). These improvements have been made in dairy cows’ production performance, healthy digestion, milk output, and immunology (18).

Although it is challenging to enhance ruminant milk with PUFA by altering the feed ration, numerous authors have found beneficial changes in the fatty acid profile of milk from cows, ewes, and goats receiving feed diets high in green forages (19). Several researchers have observed that organic milk has higher levels of MUFA, PUFA, and CLA, making it healthier and more nutritious than regular milk (19–21). According to O’Donnell-Megaro et al. (20), the mentioned variation is insufficient to impact human safety. Herbal additives may improve animal health and production. However, many phytochemical action mechanisms are unknown. In this sense, correctly identifying these phytochemicals and their appropriate doses to use them safely is essential (22). Many standardized herbal products used in animal feed have not been fully characterized yet. Therefore, the present study evaluated the effects of herbal mixture supplementation on milk production, milk quality, and some serum biochemical parameters in cows.

## 2. Materials and Methods

This study followed regulations established by Kafrelsheikh University in Egypt (Number 4/2016 EC) and approved by the local experimental animal care ethics committee.

This investigation used 30 healthy Holstein-Friesian cows with body weights (BW) of 634 ± 32.5 kg and ages 32 to 46 months, and the cows were primiparous. All the experimental cows were selected during the late before-calving stage (20 days before). According to their BW, parity, and the last milk output season, cows were separated into three groups of 10 at the beginning of the experiment. The first group was fed diets without feed additives as control, but groups two and three were fed control diets complemented by 50 and 100 g/head/day of the herbal mixture, respectively. The experiment was carried out in the summer. The temperature was 35 ± 5°C, and the relative humidity was 60–70%.

The herbal mixture used in this study was a commercial product called (Ovum)®, provided by the VESMARK company, Tanta, Egypt. The Ovum product was content 10%nuture betaine and 90% of four herbal plants (flaxseed, fenugreek, curcumin, and peppermint). The herbal mixture was delivered to the cows by being mixed with the daily feed.

Table 1 lists the ingredients and chemical composition of the total mixed ration, as the National Research Council (23, 24) recommended for dairy cows, depending on their body weight and milk production. Using the AOAC (25), a chemical analysis of typical monthly feedstuffs on a DM basis was performed.

**Table 1:** Ingredients and chemical composition of a total mixed ration.

Ingredient	% of DM	Chemical composition	% of DM
Silage	28.29	CP	15.73
Grass hay	13.28	Ca	0.75
Alfalfa hay	10.53	P	0.45
Distillers grain	10.05	NDF	39.79
Cotton seed	3.58	ADF	22.62
Extruded soybean	1.8	NE_L_, MJ/kg	6.38
Corn	18.62		
Soybean meal, 46	8.22		
Wheat bran	2.21		
NaHCO_3_	0.56		
NaCl	0.41		
Cotton meal	1.25		
Calcium phosphate	0.62		
Limestone	0.38		
Premix^1^	0.20		

1 Formulated to provide (per kg of DM) 600,000 IU of vitamin A; 110,000 IU of vitamin D; 2,150 IU of vitamin E; 4,100 mg of Zn; 30 mg of Se; 50 mg of I; 790 mg of Fe; 68 mg of Co; 1,010 mg of Mn; and 1,010 mg of Cu.

During the study period, 5 ml blood samples from the jugular vein were obtained biweekly from all cows in each group. Blood plasma was separated by centrifuging the collected blood at 15 g for 10 min, after which the plasma was stored at −20°C until chemical analysis. Blood plasma concentrations of total cholesterol, prolactin, GOT, GPT, and cortisol were measured using commercial kits by spectrophotometer (Diagnostic System Laboratories, Inc. USA) (26). Machines were used to milk Holstein cows twice daily at 5:40 and 17:30 h. Yield milk was individually noted for 120 days after calving. Milk samples were obtained weekly to determine milk composition (total fat, Triacylglycerol, cholesterol, total protein, lactose) using the Milko-Scan system (Model 133B) (23, 24, 27). The 4% fat-corrected milk (4% FCM) for each cow milk yield was determined using the following formula:

$$\begin{array}{l} 4\%\mathrm{FCM}=\mathrm{Actual milk yield}\;\left(\mathrm{kg}\right)\times 0.4+15\times \\ \mathrm{fat}\;\mathrm{yield}\;\left(\mathrm{kg}\right)\;\mathrm{Geans equation} \end{array}$$

Milk fatty acids were trans-esterified with sodium methoxide according to previously reported methods (28). Briefly, 2.0 ml of n-hexane was added to 40 μl of butter fat and vortexed for 30 s, followed by the addition of 2 ml of sodium methoxide (0.4 mol). After vortexing, the mixture was allowed to settle for 15 min. The upper phase, containing the fatty acid methyl ester (FAME), was recovered and analyzed by an Agilent 7890B Gas chromatography (GC-FID) with a polar capillary column SP R©-2,560 100 m, 0.25 mm id, 0.2 μm film thickness. Helium was used as a carrier gas at a flow rate of 20 cm sec^-1^ and split ratio of 100:1. The column temperature profile was held at 100°C for 5 min, ramp to 240°C @ 4°C min^-1^; held at 240°C for 30 min. A sample volume of 1.0 μl was injected. The FAME was identified by comparing their relative and absolute retention times with FAME standards (from C4:0 to C22:0). Fatty acid contents are presented as a percentage of total fat weight (wt%/wt%).

SPSS version 23 one-way analysis of variance (ANOVA) was used to perform the Statistical analysis followed by Duncan’s test with *p* values < 0.05. Data are expressed as a standard error means.

## 3. Result

**Figure 1:**
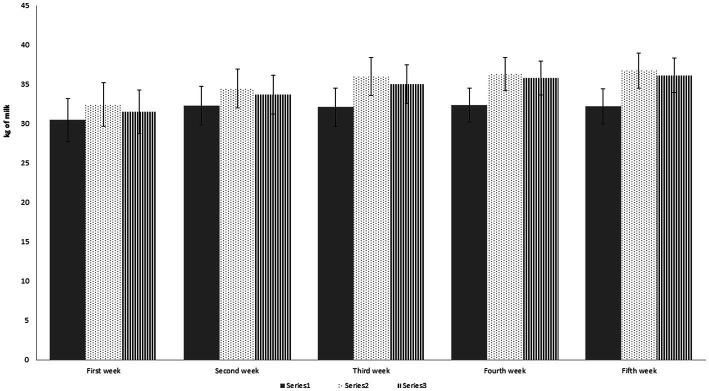
Effect of herbal mixture supplementation on milk production (Kg) per week. The amounts of milk production are represented by vertical bars. ^a,b,c^Mean values with unlike letters were significantly different (*p* < 0.05).

The results showed insignificant differences between the control group and animals that received 50 mg/head/day and those that received 100 mg/head/day Figure 1.

Concerning milk composition, the acquired results demonstrated a significant decrease (*p* < 0.05) in the animals that received Ovuma at a dose of 100 mg/head/day (173.33 ± 12.1) compared with the control group (233.33 ± 3.33). However, the control group did not differ significantly from the experimental group., which received 50 mg/head/day, or even between animals that received 100 or 50 mg/head/day (Table 2).

**Table 2:** Effect of herbal mixture supplementation on milk solid matter composition.

Indices	Control	50 mg/head/day	100 mg/head/day	p values
Total fat, %	2.64 ± 0.18	2.80 ± 0.41	3.22 ± 0.09	0.216
Triacylglycerol, mg/dl	175.93 ± 9.8	175.40 ± 21.0	168.68 ± 37.1	0.097
Cholesterol, mg/dl	233.33 ± 3.33^b^	207.66 ± 18.94^ab^	173.33 ± 12.1^a^	0.045
Total protein, mg/dl	3.36 ± 0.33	3.70 ± 0.05	3.33 ± 0.26	0.219
Lactose, mg/dl	4.53 ± 0.13^b^	4.76 ± 0.03^ab^	4.86 ± 0.03^a^	0.042

Different letters mean significant differences at *p* < 0.05. Data were represented as Mean ± SEM.

Also, lactose contents revealed a significant increase in the group that received Ovuma at 100 mg/head/day (4.86 ± 0.03) compared with the control group (4.53 ± 0.13). Meanwhile, the control group did not differ significantly from the group that received 50 mg/head/day or between animals that received 100 or 50 mg/head/day.

However, there were no appreciable variations between groups in total proteins, fat, and triacylglycerol (Table 2).

The results showed no differences between the groups concerning prolactin and cortisol levels and GPT and GOT activities. Meanwhile, plasma cholesterol showed a significant decrease in the group receiving 100 mg/head/day (193.50 ± 6.0) compared with the control group (228.25 ± 6.9). While group two, which received 50 mg/head/day, did not show a significant difference between both the control and 100 mg received group (Table 3).

**Table 3:** Effect of herbal mixture supplementation on plasma biochemical parameters.

Parameters	Control	50 mg/head/day	100 mg/head/day	*p* values
Cholesterol, mg/dl	228.25 ± 6.9^b^	209.25 ± 3.6^b^	193.50 ± 6.0^a^	0.032
Prolactin, mg/dl	3.0 ± 0.07	3.0250 ± 0.07	3.15 ± 0.08	0.167
GOT U/L	27.25 ± 0.62	26.50 ± 0.95	26.25 ± 0.85	0.164
GPT U/L	13.50 ± 0.95	12.75 ± 0.47	12.25 ± 0.47	0.113
Cortisol, mg/dl	12.75 ± 0.47	13.75 ± 0.48	13.75 ± 0.62	0.123

Different letters mean significant differences at *p* < 0.05. Data were represented as Mean ± SEM.

Concerning milk fatty acids composition, the obtained results showed that there was no significant difference between groups concerning C18, C18:1 (c9), 18:1 (c11), 18:2 (c9, c12), 18:2 (t9, t12), and CLA (c9, t11). Meanwhile, both C19:00 and 18:3 (c6, c9, and c12) were increased significantly (*p* < 0.05) in the group that received 100 mg/head/day, followed by 50 mg/head/day, compared with the control group (Table 4).

**Table 4:** Effect of herbal mixture supplementation on milk fatty acids composition.

Fatty acid	Control	50 mg/head/day	100 mg/head/day	*p* values
C18	140.0 ± 0.91	140.25 ± 1.13	141.0 ± 0.71	0.231
C18:1 (c9)	233.75 ± 2.01^c^	240.75 ± 2.04^b^	245.75 ± 3.19^a^	0.067
18:1 (c11)	18.75 ± 0.85^c^	21.50 ± 0.64^b^	23.50 ± 1.81^a^	0.087
18:2 (c9, c12)	23.25 ± 0.75	27.25 ± 0.47	30.75 ± 0.75	0.117
18:2 (t9, t12)	4.52 ± 0.51^c^	6.87 ± 0.41^b^	7.70 ± 0.53^a^	0.064
C19:00	0.63 ± 0.02^c^	0.71 ± 0.02^b^	0.79 ± 0.06^a^	0.032
18:3 (c6, c9, c12)	0.46 ± 0.005^c^	0.53 ± 0.008^b^	0.64 ± 0.003^a^	0.021
CLA (c9, t11)	5.90 ± 0.16	6.30 ± 0.27	6.42 ± 0.28	0.239

Different letters mean significant differences at *p* < 0.05. Data were represented as Mean ± SEM.

## 4. Discussion

This study confirms previous reports in dairy cattle of a non-substantial impact of the herb mixture on milk output (29–32) and buffaloes (33). Buffaloes fed a diet supplemented with garlic and peppermint showed no change in DMI or nutritional digestibility (34). Because rumen fermentation characteristics are linked to milk production (35), no further changes in milk yield characteristics were observed due to the lack of treatment effects on rumen fermentation. The impacts of phytochemical mixtures, such as cinnamaldehyde, eugenol, and capsicum, on milk production in dairy calves have also been examined, with conflicting findings (36, 37). Equally, no impact of eugenol on cows’ milk production was found (36).

According to recent research, Supplemental Betaine can boost ruminal fermentation under osmotic and thermal stress, restore trypsin and amylase’s affinity to counteract the inhibitory effects of hyperosmolarity and increase the milk yield (38). There was also no discernible difference in the productive efficiency of dairy animals when eugenol and cinnamaldehyde were combined (39).

The herbal mixture supplementation hurt milk cholesterol and positively affected milk lactose while not affecting total fat, Triacylglycerol, and protein. Compared to the control group, herb-supplemented buffalo tended to produce milk with a higher fat percentage. Milk fat content is connected to acetate and butyrate concentrations, which are precursors to numerous bodily substances, including fatty acids and total cholesterol (40, 41). In addition, their concentrations are closely related to the kinetics of fermentation in the rumen. DMI, milk yield, or conformation did not differ significantly (except milk fat) after peppermint supplementation in dairy cows, as was earlier explained (42).

The mammary gland is one of the few adult tissues that strongly induce *de novo* fatty acid synthesis upon physiological stimulation, suggesting that fatty acid is essential for milk production during lactation. The committed enzyme to perform this function is fatty acid synthase (FASN). The milk fatty acids composition is affected by herbal mixture supplementation, consistent with (43). Comparing our results to previous reports on cattle and buffaloes, different researchers find that the SFA (62–64%) and UFA (36–38%) contents are comparable (44). Milk’s desired fatty acid concentrations can be increased through dietary polyphenolic component manipulation of microbial biohydrogenation in the rumen (19). Earlier Increases in linoleic acid (up to 12%) and linolenic acid (up to 13%) in the herbal mixture were found to be comparable with previous reports of increases of close to 30% rises in these milk fatty acids in consequence of feeding sheep with fodder (condensed tannins) rich in polyphenolic (45). The herb combination’s favorable effects on human health are seen in the increase in UFA levels and the reduction in significant SFA (Stearic acid; C18:0) by approximately 10 percent. Those findings have a rational explanation due to polyphenolic chemicals selectively modulating certain microorganisms in the rumen, reducing the biohydrogenation of ingested fatty acids and increasing the fraction of UFA (46, 47). There was a positive correlation between the Butyrivibrio species’ relative abundance after treatment and the milk’s levels of linolenic acid and n-3 fatty acids (48). In addition, as was indicated above, due to their greater abundance and probable link with milkʼs fat content, additional bacterial taxa contributed to the increase in UFA levels in HM20.

Blood Cholesterol was decreased while; prolactin, GOT, GPT, and cortisol were unaffected. Total blood cholesterol levels can be influenced by both exogenous (from food and dietary supplements) and endogenous (from the body itself) factors. Saturated fatty acids in the diet raised total blood cholesterol levels, while polyunsaturated fatty acids lowered them. However, saturated fatty acids were twice as effective. Also, like humans, those fatty acids can control cattle liver’s lipid metabolism (49, 50). These three herbs, peppermint, clove, and lemongrass, have been associated with pharmacological effects (51–53). Consumption of these plants may have a suppressive impact on body functions. In the current investigation, plasma metabolite, enzyme, and hormone values were not significantly different between the herb-feeding treatments and the control group.

Conversely, we found that cholesterol levels significantly increased. These findings suggest that herb feeding does not impede the function of organs linked to plasma chemicals. However, the impact of administering herbs to cattle on how their organs develop was not evaluated over more than 2 weeks. Our findings demonstrate that herbal infusions can alter the milkʼs fatty acid profile. The amount of dietary linoleic acid (LA) and alpha-linolenic acid (ALA), the grade of ruminal biohydrogenation, and the amount absorbed in the duodenum all contribute to the final milk concentration (54). Because of the positive effects on human health, it is encouraging to see this concentration rising. For instance, alpha-linolenic acid has shown promise as a neuroprotectant, anti-inflammatory, and mood-lifting agent (18, 55). Recent research by de Goede (56) indicates that increasing ALA consumption reduces the risk of stroke. ALA is metabolized in the body to eicosatetraenoic acid, a fatty acid with cardioprotective and other human health advantages (51–53, 57).

## 5. Conclusion

Supplemental feeding of herbal preparations (OVUMA) to nursing Holsteins cows improved rumen parameters, milk output, and animal productivity in the current study. Because these treatments are non-hormonal and combine several herbs, they are safe, economical, and environmentally friendly, with no harmful consequences. Consequently, to increase the efficiency of feedstuffs, lessen the negative impacts of environmental stress, and enhance the overall performance, health, and feed cost of animals, it should be recommended to include these herbal remedies in the dairy cows’ diet.

## Data availability statement

The original contributions presented in the study are included in the article/supplementary material, further inquiries can be directed to the corresponding author/s.

## Ethics statement

The animal study was reviewed and approved by The animal care ethics committee (Number 4/2016 EC), Kafrelsheikh University, Egypt.

## Author contributions

AS, MY, and NE: supervision, project administration, conceptualization, methodology, formal analysis, supervision, and writing – original draft. MMS, HE-S, and MS: conceptualization, methodology, formal analysis, and investigation. MW, SC, IK, and HE: supervision, visualization, resources, data curation, and writing – review and editing. All authors contributed to the article and approved the submitted version.

## Conflict of interest

The authors declare that the research was conducted in the absence of any commercial or financial relationships that could be construed as a potential conflict of interest.

## Publisher’s note

All claims expressed in this article are solely those of the authors and do not necessarily represent those of their affiliated organizations, or those of the publisher, the editors and the reviewers. Any product that may be evaluated in this article, or claim that may be made by its manufacturer, is not guaranteed or endorsed by the publisher.
